# Extirpation of the Entire Lower Lip; of the Glandulœ Submaxillares and Sublinguallis; Resection of the Rami Horizontales of the Lower Maxilla, for Cancer—Cheiloplasty

**Published:** 1860-01

**Authors:** A. G. Walter

**Affiliations:** Pittsburg, Pa.


### ARTICLE XVIII.

*Extirpation of the entire Lower Lip; of the Glandulæ Submaxillares and Sublingualis; Resection of the Rami Horizontales of the Lower Maxilla, for Cancer—Cheiloplasty.* By A. G. Walter, M. D., of Pittsburg, Pa.

Nicholas Huebner, a German laborer, 63 years of age, of good constitution and youthful activity, entered my hospital on April 14th, 1859, with extensive ulceration of the lower lip, suffering excessive pain.

Being loathsome to himself and friends, he had attempted suicide, some two weeks ago, by jumping into the river, but was rescued.

The history of the case is as follows: Six months ago a wart appeared on the left angle of the mouth, which soon assumed the character of fungus granulation, spreading over the whole lip, beyond and above the angles of the mouth and over the chin. The remnant of the lip was everted, while the fungus was very prominent, covered with offensive bloody matter, and excavated in the middle, leaving the periosteum of the aveolar process, and the incisor and canine teeth of the lower jaw exposed. There was then no enlargement of the submaxillary and sublingual glands, but great pain in the diseased parts, with irritative fever. His constitution, otherwise excellent, began to feel the inroads of the cancerous infection. Relief was offered him by removal of the diseased mass, to which he willingly assented.

Assisted by several medical friends, the paient being fully etherized, the carcinomatous mass was extirpated, by incising first the angles of the mouth into the cheek, to the extent of $1\frac{1}{2}$ inches and circumscribing the fungus by two lateral elliptical cuts, which met some distance below the sympyhsis menti. The flaps of the cheek thus formed, were dissected from the lower jaw, to admit of the passing of a curved trocar behind the inner face of the maxilla, through the canula of which a chain saw was carried, and the symphysis menti obliquely resected on each side, from behind and outward, forward and inward; $2\frac{1}{2}$ inches of the maxilla was thus removed. The tongue being previously secured by an ansa, its powerful traction was plainly but harmlessly felt. There was arterial bleeding from several vessels, four of which required ligation. The flaps of the cheek meeting readily, without straining, were next approximated, and united by several strong Carlsbad needles and silver wire; an opening being left at the lower angle of the wound, for the exit of the secreta. The mucous membrane of the mouth was united to the dermis of the new upper lip, as well as to the lower one. There was a space of about an inch between the approximated extremities of the maxilla.

The patient bore the operation well. Brandy and hot coffee restored the circulation to its legitimate standard. Great difficulty of swallowing was present for some days after, the tongue having lost its attachment to the jaw. Phlegm, too, collecting around the pharynx, added greatly to his discomfort, the patient not being able to dislodge it by expectoration. Syringing the mouth at frequent intervals, however, with warm water, alleviated the pharyngeal distress, by washing away the glandular secretions of the mouth. He passed a comfortable night under the influence of an opiate, and was next morning walking about the yard of the hospital. Being sustained by brandy, broths and nocturnal anodynes, his recovery went on uninterruptedly. The difficulty of swallowing gradually subsided, as the detached muscles of the tongue formed adhesions to the newly formed chin.

In five days the wound had united by first intention, all pins being removed, as well as the ansa from the tongue, by which it was secured to the cheek ; the lower angle of the wound still discharging some matter, and the secreta of the mouth.

In thirteen days from the date of the operation, the patient left the hospital, apparently in better health, and with more strength than when he entered. A month later, a piece of bone, from the resected maxilla, was exfoliated, the fistula below the chin having healed.

Three months after, he presented himself again in excellent health, yet complaining of the appearance of a hard, movable tumor, of the size of a marble, under the lower jaw, in the mesian line, and with slight enlargement of both maxillary glands. The former rapidly increased to near the larynx, and upward, the superincumbent skin becoming incorporated, assumed a purplish hue, and was perforated by several fistulæ, with soft yellow, fungous bottom, discharging offensive matter. Return of pain, too, augmented his anxiety. The submaxillary glands gradually enlarged, the tumor of the chin being the size of a hen's egg. The resected ends of the maxilla had nearly met in the median line. His powers of mastication were perfect. Such being the condition of the patient, relief was again attempted, though the chances of success were rather slender.

On July 5th, under the influence of ether, the lower lip was divided in the median line, and the diseased mass circumscribed far beyond its outlines, by two elliptical incisions, meeting at the pomum adami of the larynx. The lateral flaps of the cheek were again separated from the maxilla, which was divided by a chain saw, one inch anteriorly of the angle of the lower jaw, at each side. The mucous membrane of the mouth next being dissected from the rami of the jaw, the whole mass was extirpated from one side to the other, down to the root of the tongue and larynx, leaving the somewhat enlarged sublingual gland intact. Both submaxillary glands were next removed, by careful dissection. The bleeding was rather active, several large arteries, and many smaller branches requiring ligation. The tongue, though secured by an ansa, did not retract. The lower lip again was closed by several Carlsbad sutures, and, the patient being weak, the extirpation of the sublingual gland, and the closure of the wound was deferred to the next day, a linseed poultice covering the wound.

Brandy, broths, and hot coffee having again sufficiently revived the vital powers, the sublingual gland was carefully extirpated, the lingual artery having been tied. The large wound left in the neck, had to be closed by transplantation. By cutting from the middle of the margin of the wound on each side outward, and curving upward to about an inch anteriorly below the lobe of the ear, and dissecting the flaps. Sufficient cutis was obtained by elongation to close half of the gap of the neck, in the median line, by Carlsbad needles. Two similar incisions, with their cornua down the sides of the neck, detached two flaps, which, on stretching, were carried in front and secured by sutures, thus closing the wound completely. The denuded portions of the sides of the face and neck were left bare, a cataplasma covering the whole; the chin being approximated to the breast, and confined in this position.

The patient arose from the operating table with no evident mark of exhaustion. Strange as it may appear, no untoward reaction followed. An opiate procured rest at night; brandy and beef tea soon restored him; ptyalism, so troublesome after the first operation, was wanting; deglutition not being impeded. The wound healed rapidly by first intention, only a small opening remaining at the root of the tongue; the side wounds of the face and neck closing by granulation and cicatrization. The patient left his bed daily, and had gained strength enough in three weeks to leave the hospital, the wounds being closed, with the exception of a small opening below the tongue. Remarkably displayed, in this instance, was the rapidity of plastic adhesion. The patient presented himself frequently, is still well, and entertains strong hopes of having conquered his enemy. The deformity of the face, as seen by the accompanying sketch, is trifling, and the power of deglutition and speech unimpaired, the loss of the submaxillary and sublingual glands not being felt.

There being no part of the human body which bears the inroads of bold surgery with greater, or even equal impunity than the face, an instructive lesson is thus given to the surgeon, encouraging him to extend his incisions still *further* beyond the diseased tissues than is usual, for experience has amply proved that the return of cancerous diseases, and all those of a malignant character, stands in direct proportion, not only to the *delay* of the operation, but more frequently to the *parsimoniousness* with which contiguous structures are saved, for fear of extensive mutilation. In case of cancer of the foot, it not being prudent and safe to amputate the leg, but the femur ; so in operations on the face for malignant diseases, the incisions, excisions and resections must reach *far*, *far* beyond the diseased outlines.

How long the patient may remain free from a return of his disease is doubtful ; still there is hope that success may remain perfect, as nearly five months have elapsed since the second operation. But even if failure, at a future day, should mar the pleasure of our efforts at relief, the interest in the progress of the present case is not lessened in the least, as the power of endurance and recuperation of the system, far exceeding what reasonably could have been expected in a patient so advanced in years, have been plainly and most strikingly portrayed.

[*Med. Surg. Reporter.*

